# Neuronally-enriched exosomal microRNA-27b mediates acute effects of ibuprofen on reward-related brain activity in healthy adults: a randomized, placebo-controlled, double-blind trial

**DOI:** 10.1038/s41598-022-04875-y

**Published:** 2022-01-17

**Authors:** Kaiping Burrows, Leandra K. Figueroa-Hall, Rayus Kuplicki, Jennifer L. Stewart, Ahlam M. Alarbi, Rajagopal Ramesh, Jonathan B. Savitz, T. Kent Teague, Victoria B. Risbrough, Martin P. Paulus

**Affiliations:** 1 Laureate Institute for Brain Research, 6655 South Yale Ave, Tulsa, OK 74136 USA; 2 Department of Community Medicine, University of Tulsa, Tulsa, OK USA; 3 Departments of Surgery and Psychiatry, School of Community Medicine, The University of Oklahoma, Tulsa, OK USA; 4 Department of Pathology, University of Oklahoma Health Sciences Center, Oklahoma City, OK USA; 5 Department of Biochemistry and Microbiology, The Oklahoma State University Center for Health Sciences, Tulsa, OK USA; 6 Department of Pharmaceutical Sciences, The University of Oklahoma College of Pharmacy, Oklahoma City, OK USA; 7 Center of Excellence for Stress and Mental Health, La Jolla, CA USA; 8 Department of Psychiatry, University of California, San Diego, La Jolla, CA USA

**Keywords:** Magnetic resonance imaging, Randomized controlled trials, Reward, Cellular neuroscience

## Abstract

This double-blind, randomized, within-subjects design evaluated whether acute administration of an anti-inflammatory drug modulates neuron-specific, inflammation-modulating microRNAs linked to macroscopic changes in reward processing. Twenty healthy subjects (10 females, 10 males) underwent a functional magnetic resonance imaging scan while performing a monetary incentive delay (MID) task and provided blood samples after administration of placebo, 200 mg, or 600 mg of ibuprofen. Neuronally-enriched exosomal microRNAs were extracted from serum and sequenced. Results showed that: (1) 600 mg of ibuprofen exhibited higher miR-27b-3p, miR-320b, miR-23b and miR-203a-3p expression than placebo; (2) higher mir-27b-3p was associated with lower insula activation during MID loss anticipation; and (3) there was an inverse relationship between miR-27b-3p and MID gain anticipation in bilateral putamen during placebo, a pattern attenuated by both 200 mg and 600 mg of ibuprofen. These findings are consistent with the hypothesis that miR-27b could be an important messaging molecule that is associated with regulating the processing of positive or negative valenced information.

## Introduction

Inflammatory processes may play a significant role in psychiatric disorders in general^1^, and mood disorders in particular^2^. Epidemiological data suggest an overlap between depression and inflammatory illnesses^3,4^, and prospective studies show that chronic inflammation increases depression risk^5^. One core feature of a major depressive episode is anhedonia—loss of interest or pleasure in nearly all rewarding activities. Previous research suggests that elevated peripheral inflammation is associated with decreased brain striatal activity during reward prediction and anticipation in major depressive disorder (MDD)^6,7^. Here, we investigate whether ibuprofen, a non-steroidal, anti-inflammatory drug (NSAID), will alter neuronally-enriched exosome (NEE), inflammatory-related microribonucleic acids (miRs), and modulate reward-related brain processing in healthy participants.

Clinical trials have investigated the antidepressant effects of NSAIDs, including selective cyclooxygenase (COX) inhibitors such as ibuprofen, given their strong anti-inflammatory properties^8,9^. While ibuprofen is one of the most used and prescribed NSAIDs, few research studies have examined ibuprofen’s effect on the brain in human participants using neuroimaging, and those that have, exclusively focused on pain-related processing. In the double-blind, placebo-controlled, randomized, cross-over pharmacological functional magnetic resonance imaging (phFMRI) study by Pizzi et al., ten healthy subjects underwent a painful somatosensory stimulation of the right median nerve, which led to a task-related increase of blood oxygen level dependent (BOLD) signal between drug and placebo in the primary somatosensory area that was not related to changes in subjective pain scores^10^. Another study using ibuprofen (600 mg single dose) failed to suppress the secondary mechanical hyperalgesia-evoked neural response in a region of the brainstem's descending pain modulatory system and left posterior insular cortex and secondary somatosensory cortex^11^. Thus, while there is some evidence of pain-related processing changes in the brain, there is no study examining the effects of ibuprofen on reward-related processing, investigating ibuprofen’s ability to modulate reward-related brain processing, particularly in the context of ibuprofen-mediated changes in miR expression, may elucidate potential treatment targets for depressed subjects with high inflammation to improve their anhedonic symptoms.

**Figure 1:**
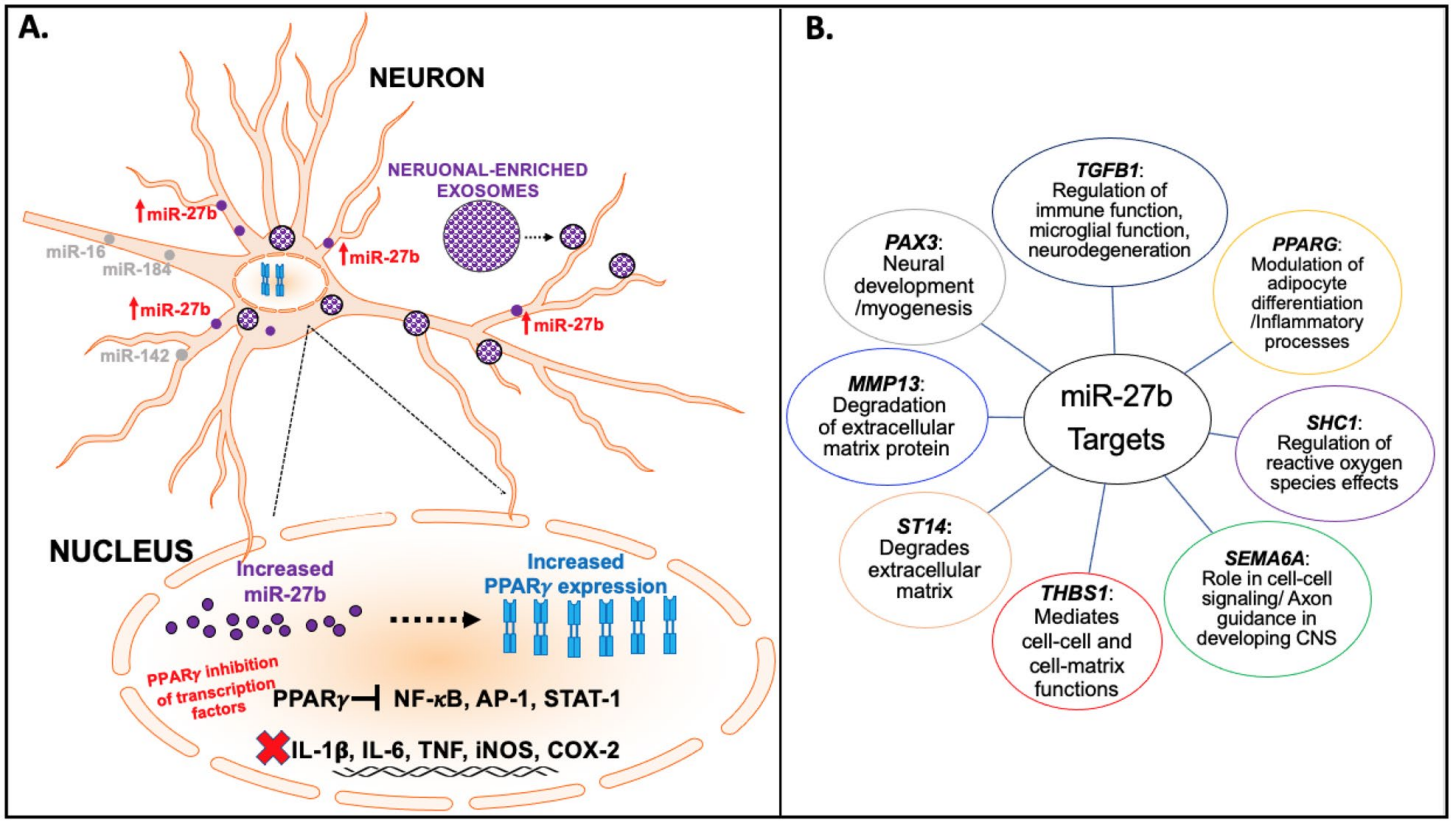
Regulatory role and targets of miR-27b. Neuronally-enriched exosomes (NEEs) are shown to express miRNAs for intracellular communication and aberrant expression in various health conditions. (**A**) Significant increases in NEEs mir-27b can lead to regulation of several targets including increased expression of PPARγ, which inhibits the expression of several transcription factors, such as NF-κB and AP-1, leading to the attenuation of proinflammatory mediators including IL-6 and TNF. (**B**) Other miR-27b gene targets include PAX3, TGFB1, PPARG, SHC1, MMP13, ST14, THBS1, and SEMA6A that mediate and regulate several functions including neuronal development, inflammatory processes, degradation of extracellular matrix, and cell–cell functions. *PPARγ* peroxisome proliferator-activated receptor gamma, *NF-kB* nuclear factor kappa-light-chain-enhancer of activated B cells, *AP-1* activator protein 1, *STAT-1* signal transducer and activator of transcription 1, *IL-1B* interleukin-1 beta, *IL-6* interleukin-6, *TNF* tumor necrosis factor, *iNOS* inducible nitric oxide synthase, *COX-2* cyclooxygenase 2, *TGFB1* transforming growth factor beta 1, *SHC1* Src homology 2 domain containing transforming protein 1, *SEMA6A* semaphorin 6A, *THBS1* thrombospondin 1, *ST14* suppressor of tumorigenicity 1, *MMP13* matrix metallopeptidase 13, *PAX3* paired box 3.

MiRs comprise a large family of small, non-coding RNAs that act as key posttranscriptional regulators of gene expression. miR-27b is significantly expressed in the brain, upregulates neuronally expressed targets and plays a role in both excitatory and inhibitory neurotransmission through the modulation of genes involved in regulation of glutamate and gamma aminobutyric acid metabolism and transport^12^. miR-27b also attenuates inflammatory mediators, such as nuclear factor kappa-light-chain enhancer of activated B cells (NF-κB) and interleukin (IL)-6^13^, with modulation of targets including peroxisome proliferator-activated receptor gamma (PPARγ and PPARα^14^ a subfamily of nuclear receptor proteins and key regulators of inflammation, which may also be potential targets for ibuprofen as depicted in Fig. 1A. miR-27b also regulates other gene targets, which have roles in inflammatory processes, extracellular matrix degradation, and cell–cell signaling as shown in Fig. 1B.^15^

**Figure 2:**
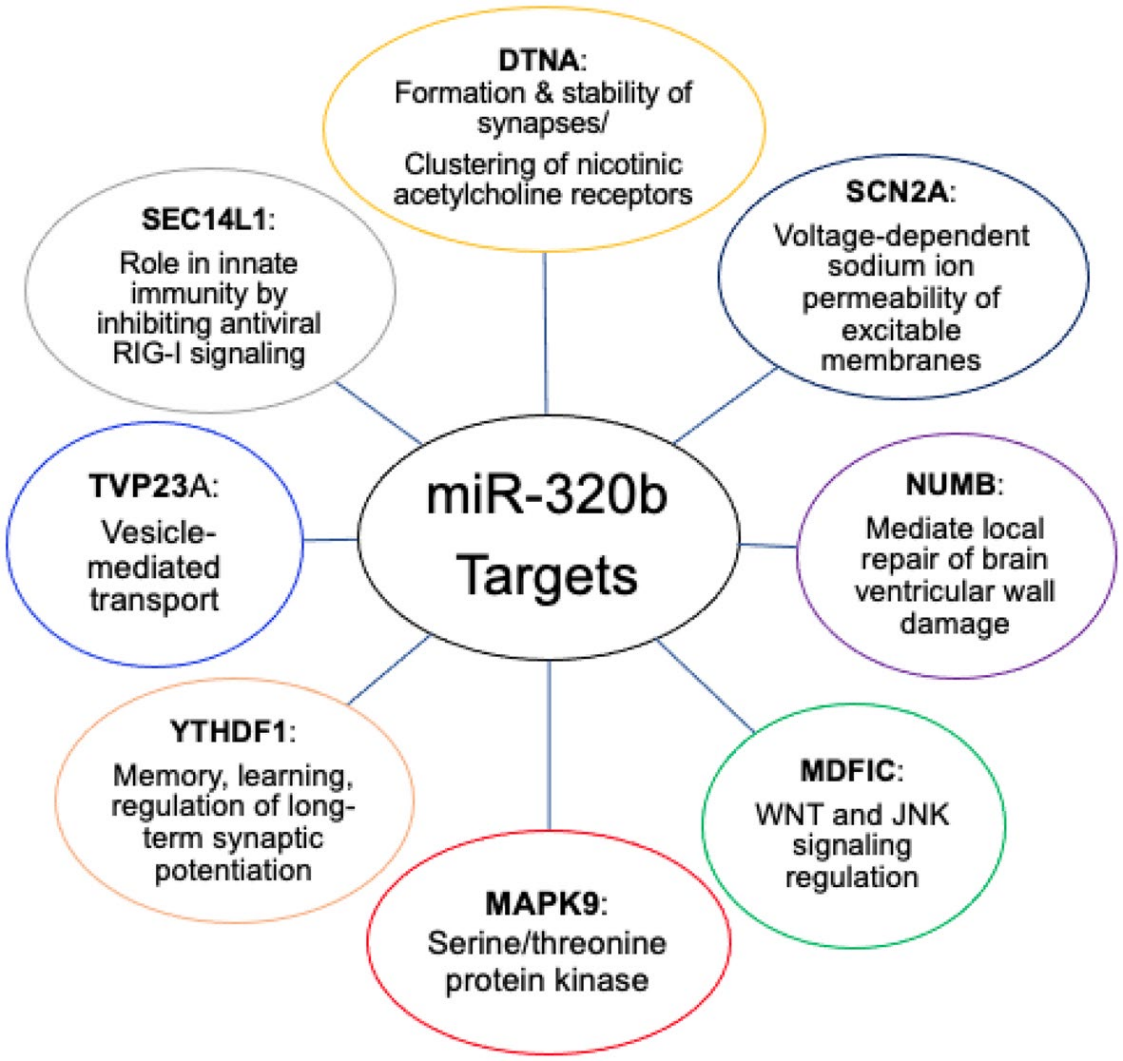
miR-320b gene targets and functions. miR-320b acts on various gene targets including DTNA, SCN2A, NUMB, MDFIC, MAPK9, YTHDF1, TVP23A, and SEC14L1. These genes mediate several functions including formation and stability of synapses, local repair of brain ventricular wall damage, WNT and JNK signaling, serine/threonine protein kinase activation, memory, learning, and long-term synaptic potentiation, vesicle-mediated transport and innate immunity through RIG-I signaling. *DTNA* dystrobrevin alpha, *SCN2A* sodium voltage-gated channel alpha subunit 2, *NUMB* NUMB endocytic adaptor protein, *MDFIC* MyoD family inhibitor domain containing protein, *MAPK9* mitogen-activated protein kinase 9, *YTHDF1* YTH N6-methyladenosine RNA binding protein 1, *TVP23A* trans-golgi network vesicle protein 23 homolog A, *SEC14L1* SEC14 like lipid binding 1, *WNT* wingless-related intergration site, *JNK* c-jun N-terminal kinase.

In comparison, miR-320b is also expressed in the brain and has been implicated in neuronal differentiation^16,17^, regulation of inflammatory processes via the nucleotide-binding oligomerization domain^18^, and regulation of endoplasmic reticulum stress^19^. miR-320b is robustly increased in anterior cingulate cortex and habenula of individuals with MDD^20^, regions of the brain thought to be important for pain affect and processing^21,22^. Figure 2 shows additional gene targets for miR-320b, which are responsible for functions such as formation and stability of synapses, local repair of brain ventricular wall damage, vesicle-mediated transport, and innate immune-mediated antiviral inhibition^23^. Both miR-27b and miR-320b affect a range of metabolic, inflammatory, and pain processes and are, in turn, modulated by anti-inflammatory drugs such as ibuprofen. These miR molecular signatures and miR-mediated functions can be experimentally investigated through the enrichment of extracellular vesicles (EVs), with a focus on brain-specific EVs.

**Figure 3:**
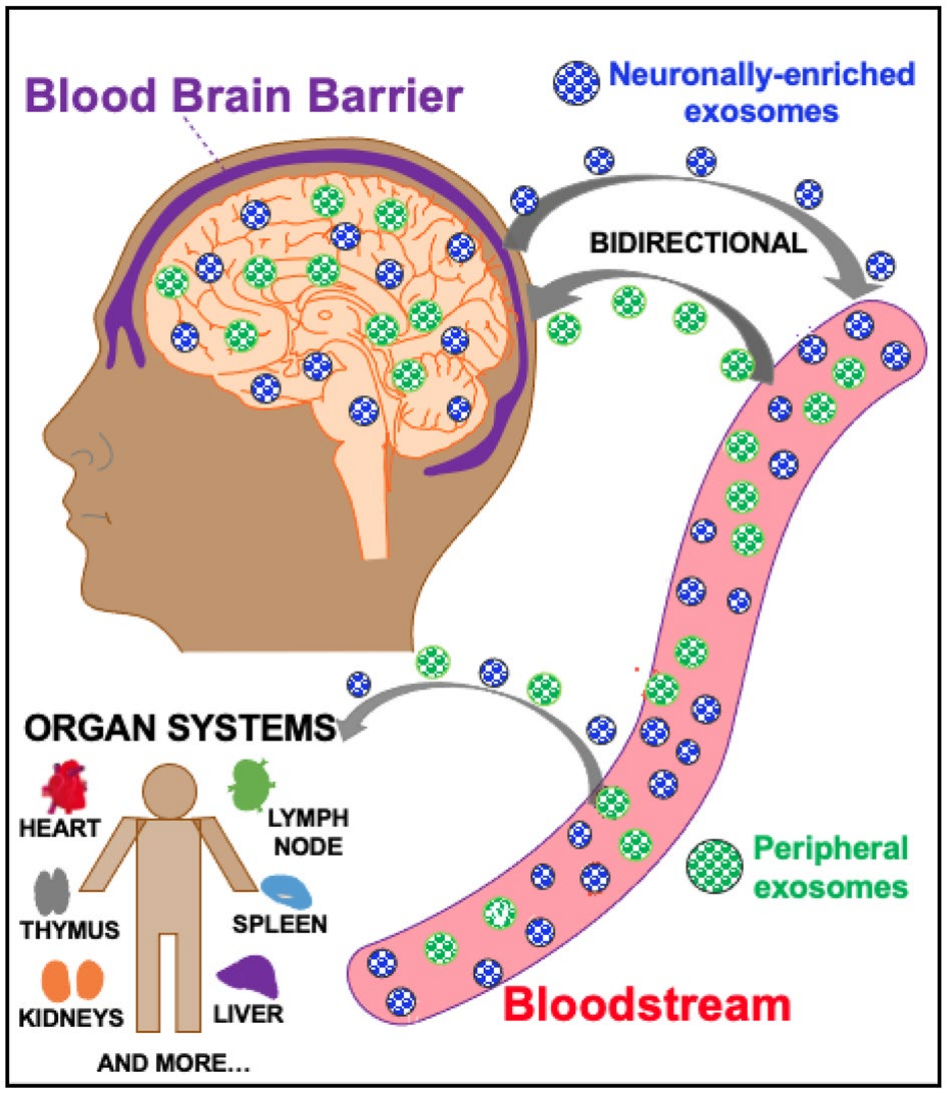
Neuronally-enriched exosomal pathway. NEEs (blue) are released from neurons in the central nervous system (CNS) and cross the blood brain barrier (BBB) into the bloodstream. This allows for NEEs capture from platforms including serum and plasma. Peripheral exosomes (purple) can also cross the BBB into the CNS to mediate a bidirectional pathway. Exosomes in the bloodstream also travel to different organ systems in the body including the heart, liver, kidneys, etc.

EVs, comprised of a diverse set of particles including exosomes, mediate complex and coordinated communication among neurons, astrocytes, and microglia, both in the healthy and diseased brain^24^. NEEs in the central nervous system (CNS) cross the blood brain barrier^25^ into the blood (Fig. 3) and peripheral exosomes from the blood and organ systems (heart, liver, and thymus, among others) also cross into the CNS. Emerging evidence suggests that miRs make up a high percentage of the specialized cargo contained in EVs and mediate their biology, function, and therapeutic potential^26,27^. Additionally, miRs are an important component in regulating critical pathways in the brain associated with MDD, amyotrophic lateral sclerosis, Alzheimer's disease, and Parkinson's disease, which share several neuroinflammatory-associated processes^28^. While brain-specific EVs from psychiatric and neuroinflammatory conditions can point to dysregulated molecular signatures and pathways, the experimental investigation of brain-specific EVs in healthy controls can also give insight into homeostatic processes and changes mediated by administration of anti-inflammatory pharmacologics.

This investigation aimed to integrate several experimental approaches to provide evidence that an acute administration of an anti-inflammatory drug modulates EV cargo, and more specifically, NEE-specific, inflammatory-modulating miRs that are linked to macroscopic changes in brain function – reward processing, in particular. Specifically, we conducted a pharmaco-phfMRI study of acute administration of placebo, 200 mg (mg), or 600 mg ibuprofen in healthy volunteers during the monetary incentive delay (MID) task^29^. Subsequently, we isolated exosomes from blood samples, enriched them for NEEs, and performed an RNAseq analysis focusing on miRs implicated in ibuprofen-regulated pathways. Lastly, we conducted a moderator analysis to determine whether the brain activation changes induced by ibuprofen were modulated by the level of miRs obtained from NEEs. Given the link between inflammatory mediators and reward processing^7,30,31^, we hypothesized that inflammatory pathway-related miRs moderate ibuprofen-related brain activation changes in striatal regions.

## Results

### NEEs MiR results

**Figure 4:**
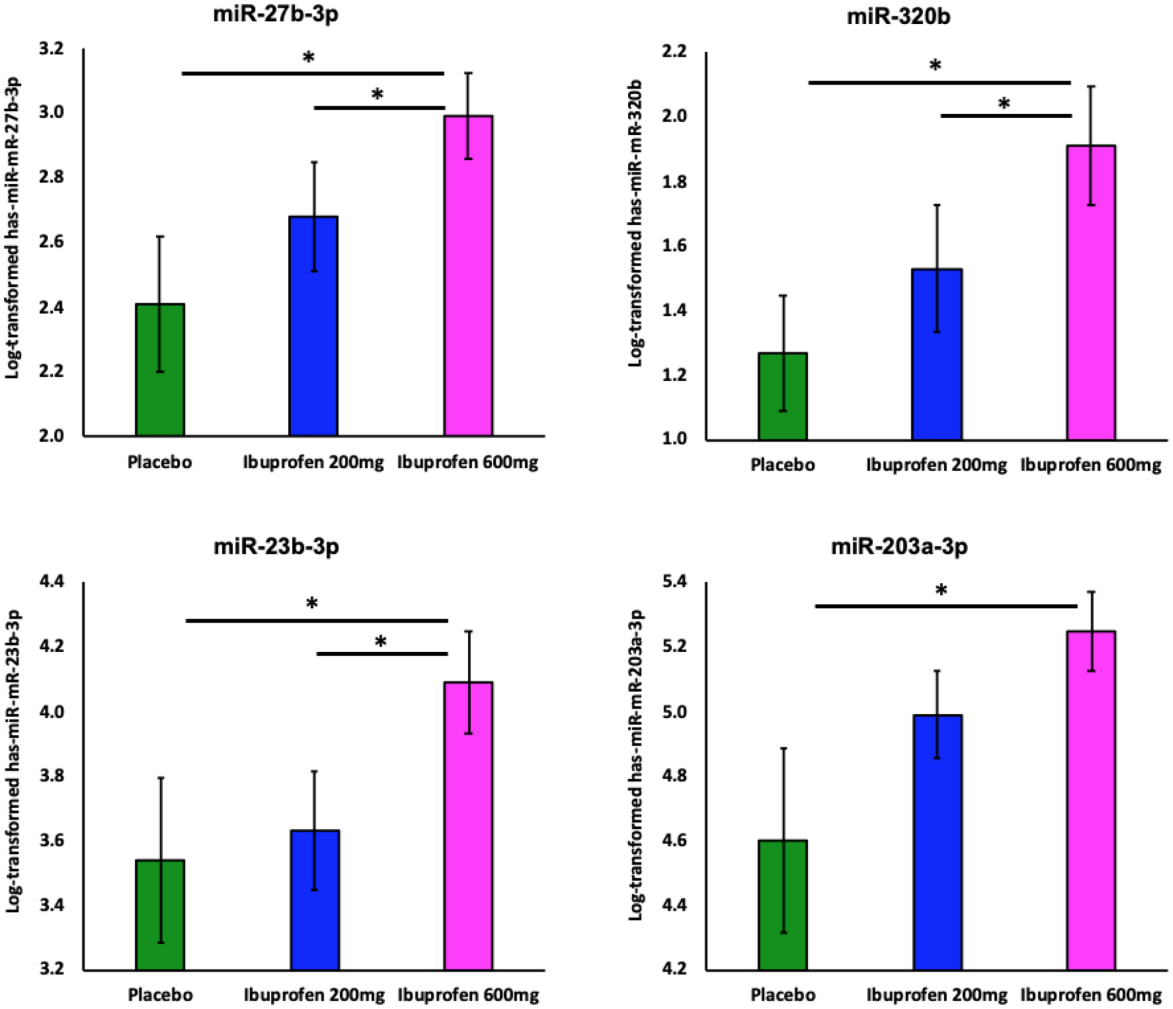
Ibuprofen-mediated effect on NEEs MiRs (n = 20). Log-transformed data shows ibuprofen-mediated (200 and 600 mg) effects on neuronally-enriched exosomal miRNA expression for (**A**) miR-27b-3p; (**B**) miR-320b; (**C**) miR-23b-3p. Only 600 mg ibuprofen differed for (**D**) miR-203a-3p. * denotes significant differences between groups using paired t-test.

There was a significant main effect of ibuprofen dose on miR-27b-3p (F_1_,_18_ = 5.38, *p* = 0.015), miR-320b (F_1_,_18_ = 6.69, *p* = 0.007), miR-23b-3p (F_1_,_18_ = 5.94, *p* = 0.010), and miR-203a-3p (F_1_,_18_ = 3.88, *p* = 0.040) (Fig. 4). Pairwise comparisons indicated that ibuprofen 600 mg exhibited higher expression on: (1) miR-27b-3p (*p* = 0.014, *d* = 0.746, 95% CI 0.133–1.034), miR-320b (*p* = 0.002, *d* = 0.787, 95% CI 0.269–1.006), miR-23b-3p (*p* = 0.041, *d* = 0.580, 95% CI 0.026–1.072), and miR-203a-3p (*p* = 0.037, *d* = 0.658, 95% CI 0.045–1.251) than placebo; and (2) miR-27b-3p (*p* = 0.046, *d* = 0.469, 95% CI 0.006–0.624), miR-320b (*p* = 0.048, *d* = 0.445, 95% CI 0.004–0.746) and miR-23b-3p (*p* = 0.017, *d* = 0.609, 95% CI 0.092–0.836) than ibuprofen 200 mg. See Supplemental Table S1 for a list of other MiRs detected in NEEs.

### Neuroimaging results

#### Main effect of miR-27b-3p for MID loss anticipation in bilateral insula

**Figure 5:**
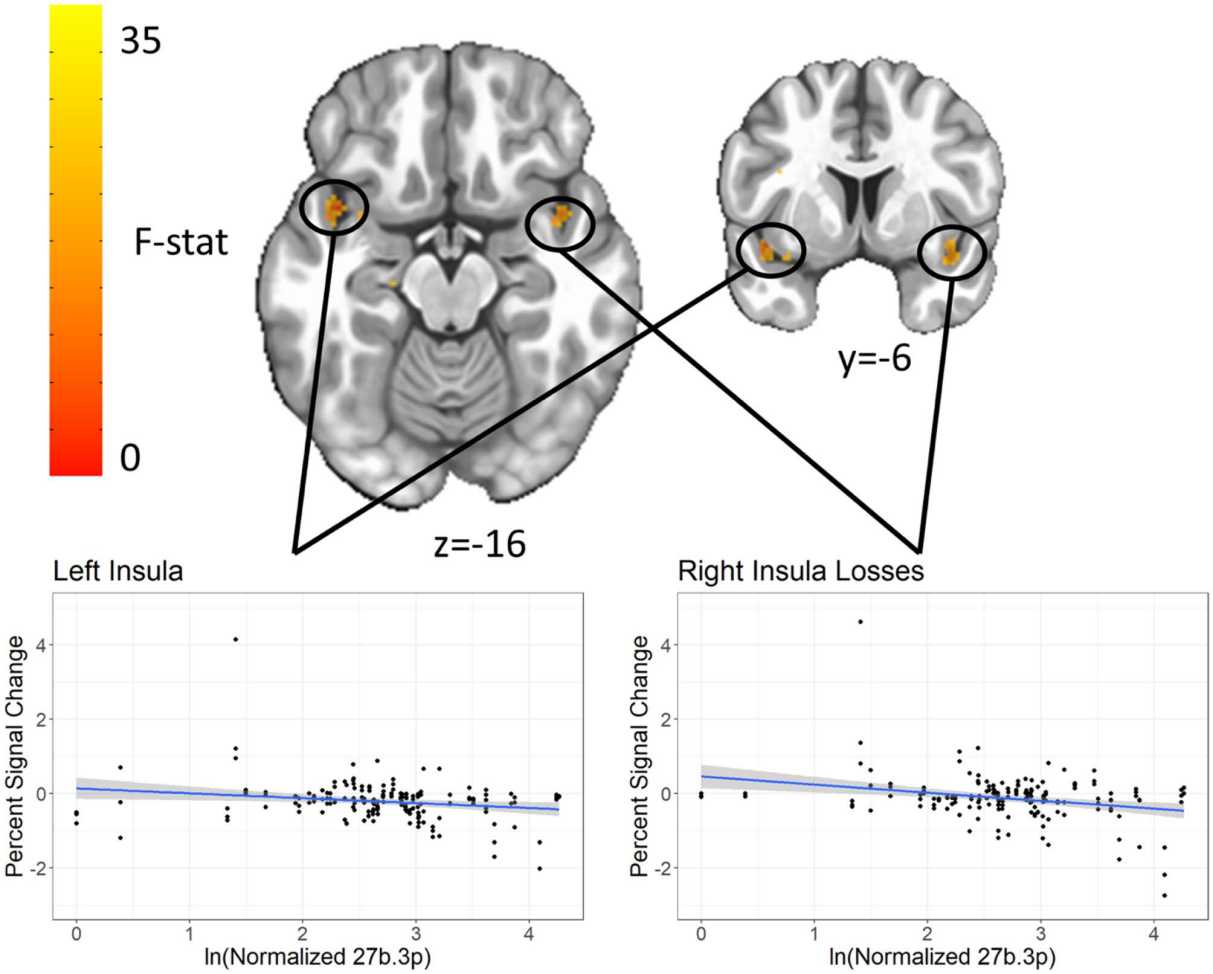
MiR-27b-3p main effect in bilateral insula for loss anticipation. Both the left and right ventral anterior insula show a main effect of levels of miR 27b-3p in response to losses, where increased miR was related to decreased activation. Clusters were extracted with a voxelwise p < 0.001 and clusterwise α < 0.01.

Both the left (Center-of-mass = 41, − 9, − 15, 156 voxels, standardized β = − 0.36, p = 0.001) and right (Center-of-mass = − 41, − 5, − 11, 62 voxels, standardized β = − 0.0.31, p = 0.004) insula showed a significant main effect of miR-27b-3p on activation during loss anticipation trials, with increased levels of miR-27b-3p related to decreased percent signal change (Fig. 5). Repeating the voxelwise analysis after removal of one outlier attenuated the effect, producing smaller clusters (75 voxels on the left, α < 0.01 and 18 voxels on the right, α > 0.1). There was no significant MiR by condition interaction.

#### Interaction between miR-27b-3p and condition for MID gain anticipation in bilateral putamen

**Figure 6:**
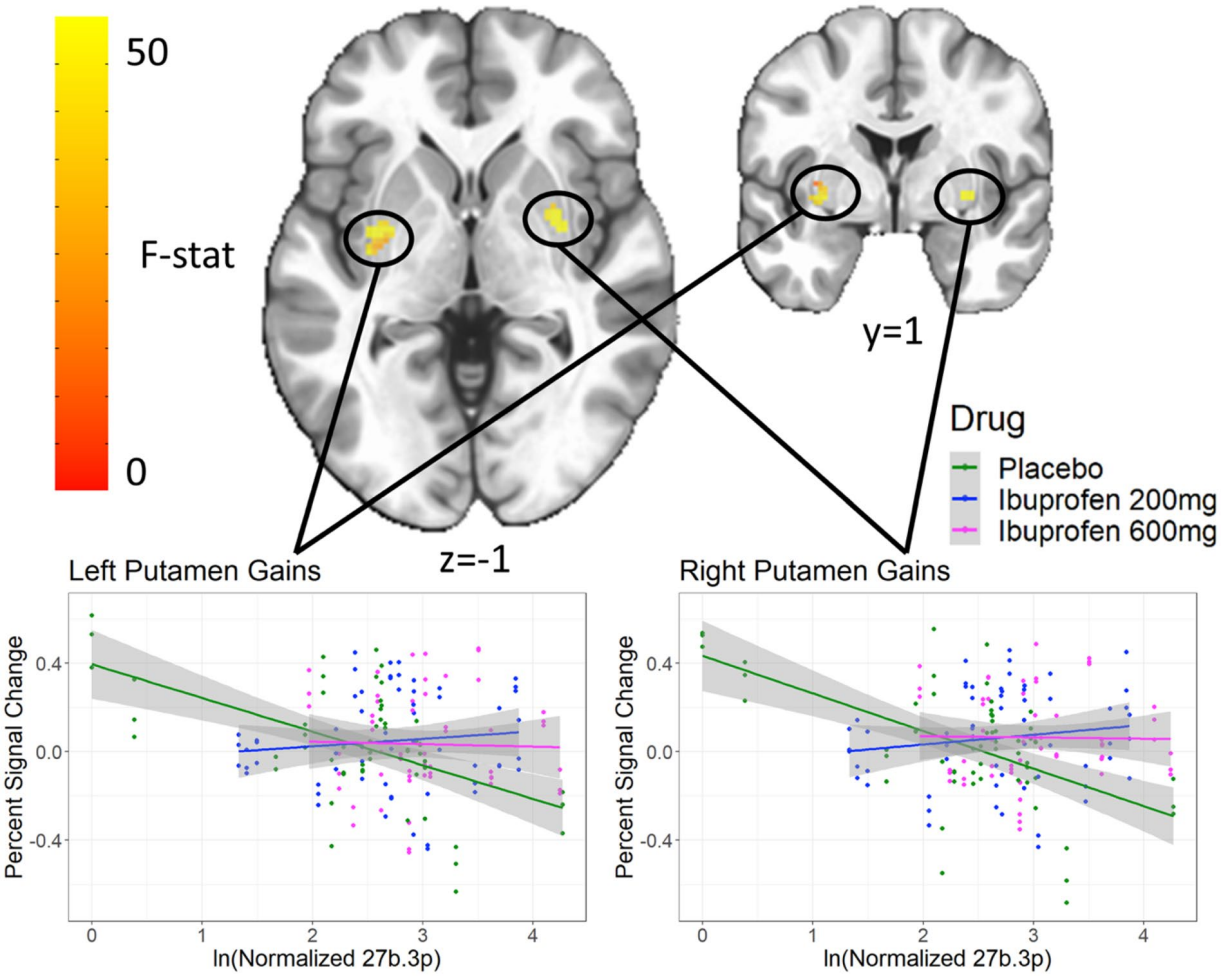
miR-27b-3p by drug interaction in bilateral putamen for gain anticipation. Both the left and right putamen show an interaction between levels of miR 27b-3p and drug in response to gains. Clusters were extracted with a voxelwise p < 0.001 and clusterwise α < 0.01.

Both the left (Center-of-mass = 31.5, 6.1, − 2.9, 89 voxels) and right (Center-of-mass = − 29.1, − 2.7, 1.2, 80 voxels) putamen showed a significant interaction between miR-27b-3p and condition on activation during the MID task during gain anticipation (Fig. 6). Post-hoc analyses showed the negative relationship between miR-27b-3p and activation in the putamen during placebo (standardized β = − 0.21, p = 0.02) was attenuated by both 200 mg (interaction standardized β = 0.32, p = 6.7 × 10^–5^) and 600 mg (interaction standardized β = 0.16, p = 0.02) of ibuprofen.

#### Other effects of miR-27b-3p and miR-320b

Several other regions showing either a main effect of miR or a miR by condition interaction are listed in Supplemental Tables S2 through S5.

## Discussion

This investigation examined the effect of an acute administration of ibuprofen on miRs modulating inflammation-related regulatory processes identified from NEEs with RNA seq analysis, and their effects on CNS function as examined by fMRI during anticipation of rewards and losses. There were three main results. First, ibuprofen dose-dependently increased miR-27b-3p, miR-320b, miR-23b-3p and miR-203a-3p, which are important modulators of various aspects of brain function. Second, higher miR-27b-3p expressions were linked to lower bilateral ventral insula responses within the context of loss anticipation. Third, ibuprofen moderated the effect of miR-27b-3p on the anticipation of gains in bilateral putamen such that both the 200 mg and 600 mg doses elicited positive correlations between miR-27b-3p and putamen activation during anticipation of gains, wherein the placebo dose elicited a negative correlation. Taken together, these findings support the notion that ibuprofen’s effects in the brain are related to modulatory miRs that can blunt the relationship between the degree of inflammation and reward-related processing.

These findings have several implications for the understanding of how anti-inflammatory drugs may affect the brain and how these brain changes may contribute to altered processing of positive or negative-valenced stimuli. The MID task has been used extensively to examine reward and loss related processing in individuals with mood or anxiety disorders^32^. Specifically, individuals with depression show an attenuation of reward-related anticipation during an episode^33^ but not when remitted^34^ and may normalize after treatment^35^. Moreover, increased levels of peripheral inflammatory markers are associated with relatively lower reward-related activation^36^, which is consistent with the view that inflammatory cytokines adversely affect positive valence processing^31^. The findings from this study suggest that one possible molecular mechanism by which inflammation may affect reward-related processing is via the signaling of inflammation-modulating miRs contained in NEEs. However, the current results further suggest that this effect is not necessarily limited to reward related processes but extends also to the degree to which the brain processes loss events. Moreover, the imaging findings support the notion that these effects are not non-specific but involve brain structures that are important for gain or loss processing, specifically, insula and putamen that are robustly activated for both gain and loss anticipations during the MID task^37^. One target with an important role on brain and inflammation are PPARs, transcription factors that affect attenuating degenerative processes in the brain and are associated with control of anti-inflammatory mechanisms^38^. Several human studies have been conducted to examine the effect of PPARγ agonists on mood^39^, including a double-blind placebo-controlled study, which showed that pioglitazone, a PPARγ agonist improved mood in MDD^40^. The ibuprofen-induced attenuation of the relationship between individuals with relatively higher levels of miRs targeting the PPAR system and brain activation during anticipation of gains or losses may provide some mechanistic explanation of how anti-inflammatory agents might partially be able to rescue attenuated brain activation in the presence of high levels of inflammation.

However, the effects of miRs may not necessarily be limited to inflammatory-related processes. As shown in Figs. 1 and 2, both miR-27b-3p, miR-320b have multiple targets that can have modulatory effects on brain function. This is consistent with findings from case–control studies in anxiety or depression showing evidence for an increase in miRs including miR-27b-3^41^ and miR-320b^20^, which has been more closely related to metabolic dysfunction^42,43^. For example, global upregulation of miR-320 has been associated with impaired gluconeogenesis, lipid metabolism, and relatively higher expression of inflammation markers^44^. The integration of functional neuroimaging studies with molecular assessments using miRs obtained from NEEs open the possibility of identifying novel, targetable disease-modifying processes in mood or anxiety disorder. Disease modifying processes can be based on circuits, behavior, or other units of analysis, which—when modulated—change the risk for, severity of, or recurrence of a disease such as mood and anxiety disorders. The use of molecular tools enables one to determine whether systems are affected that can be targeted by a pharmacological intervention. The findings from this study provide evidence that identifiable, neuronally exosomal-enriched regulatory miRs are promising targets for modulating reward and loss related processing in both subcortical and cortical brain regions.

This study has several limitations. First, the participants were healthy volunteers with no symptoms of anxiety or depression. Therefore, further studies are needed to assess the relationship between the molecular effects of ibuprofen and changes in self-reported symptoms. Second, although we observed acute changes in NEE miRs associated with inflammation-related regulatory processes, it is not clear that these changes are sufficient to be therapeutic targets or whether these changes can be maintained with chronic dosing. Third, ibuprofen had limited direct effects on reward or loss related processing in this study; thus, future investigation may focus on other anti-inflammatory modulators that may have more profound effects on brain processing. Fourth, we were unable to assess other contents within the NEEs, which may provide more direct evidence for pathways involved in the ibuprofen-mediated effects in the brain. Nevertheless, this study provides sufficient proof-of-concept evidence and delineates a limited number of molecular processes that affect the brain’s response to rewards or losses.

## Conclusions

The results from this study are consistent with the hypothesis that miR in NEE could be important messaging molecules that are altered by anti-inflammatory drugs and modify the processing of positive or negative valenced information. In this case, ibuprofen seems to attenuate the inverse relationship between inflammatory processes and reward-related activation in healthy volunteers.

## Methods

### Trial design

#### Participants

This study was conducted at the Laureate Institute for Brain Research (LIBR) in Tulsa, Oklahoma (OK) between 6/30/2015 and 10/30/2015. The study was approved by the Western Institutional Review Board (ID—LIBR # 2015-007-00), and all experiments were performed in accordance with the Declaration of Helsinki; Informed consent was obtained from all participants. Participants were recruited from the general community through newspaper, flyer, radio and other media advertisements in Tulsa and the surrounding regions of OK. Subjects were screened by trained clinical interviewers to evaluate the following study exclusion criteria: (1) history of any mental health disorder such as dysthymia, simple phobia, MDD, obsessive compulsive disorder or panic disorder as a primary diagnosis currently or within 6 months prior to the screening visit; (2) history of schizophrenia, schizoaffective disorder, or a bipolar disorder; (3) current or past 6-month alcohol or drug abuse; (4) regular use (> 15 days for past 30 days) of NSAIDS; (5) history of clinically significant hepatic cardiac, renal, neurologic, cerebrovascular, metabolic, gastric, or pulmonary disease; (6) past-year use of psychotropic drugs or antidepressants; (7) history of seizure disorders (except for childhood febrile seizures); (8) serious suicidal ideation or behavior; (9) women currently pregnant or planning to become pregnant within the next 18 weeks; (10) women currently menstruating; (11) claustrophobia, or phobia for injections or blood; and (12) fMRI-related exclusion criteria (e.g., medications treating cardiovascular, respiratory, endocrine and neurological diseases likely to influence cerebral blood flow). The trial was stopped after completing target recruitment. Twenty subjects (10 females, 10 males; mean age = 32 years, SD = 7, range = 27 to 51; mean body mass index [kg/m^2^] = 27, SD = 6, range = 20.4 to 44.7) completed this study (Table 1) (two additional subjects withdrew prior to study completion). See CONSORT 2010 Flow Diagram for complete consort diagram. The sample size was determined based on prior ph-fMRI studies (e.g., Aupperle et al.^45^), and a post-hoc power analysis using the R package WebPower showed that we should have 80% power to detect large (f > 0.72) effects with 20 participants each sampled three times.
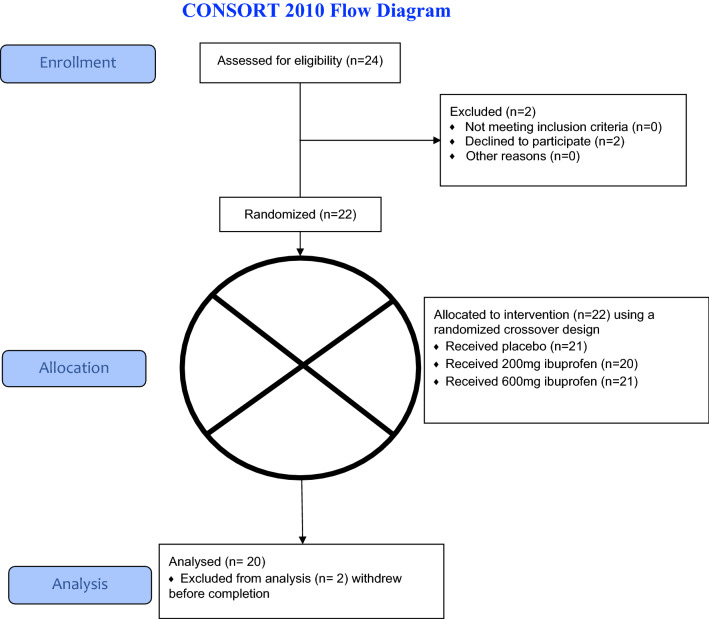

**Table 1:** Sample characteristics.

Variable	
*N*	20
Age (years)	32.4 ± 6.7
Sex = Male (%)	10 (50.0)
Body Mass Index (kg/m^2^)	26.6 ± 5.8
**Ethnicity**	
Hispanic or Latino	2
Not Hispanic or Latino	18
Refused/do not know	0/0
**Educational status**	
Grandes 1–11	0
12th grade (no diploma)	0
Regular high school diploma	0
GED or alternative credential	0
Some college, no degree	3
Associate degree	11
Bachelor’s degree	2
Master’s degree	2
Missing data	2
**Concomitant medication**	
Birth Control	3
OTC allergy medications	3
Phentermine	1
Clonazepam	1
Levothyroxine	1

#### Study outcomes

The present study of NEEs was a secondary analysis not included in the original pre-specification. The primary outcome measures for this study were the levels of NEEs miR-27b and miR-320b, with BOLD fMRI activity during the MID task as a secondary outcome. The goals were to quantify (1) the effect of ibuprofen administration on levels of NEEs (2) the effect of NEEs on reward processing, and (3) the interaction between ibuprofen administration and the effect of NEEs on reward processing.

#### Study procedures

This double-blind, randomized, cross-over study was registered on clinicaltrials.gov (Identifier: NCT02507219, Study of Ibuprofen Effects on Brain Function, first posted date: 07/23/2015). After the screening visit (T0), eligible subjects were tested three times (T1, T2 and T3) and at each visit received a single placebo, 200 mg or 600 mg dose of oral ibuprofen (dose order was counterbalanced across subjects, so that all participants received a total of 3 different doses). Ibuprofen and visually identical placebo capsules were produced by a local compounding pharmacy in Tulsa, OK. The random allocation sequence was generated using a random number generator by a statistician not involved in data collection. Drugs were labelled A/B/C and all study personnel were blinded until after data collection was complete. On the day of sessions T1, T2 and T3, subjects fasted overnight and arrived in the morning and received a snack (selected from a cheese stick, granola bar, or peanut butter crackers) along with either placebo, 200 mg or 600 mg of ibuprofen. Subjects underwent an fMRI scan approximately 1 h after dosing and a blood draw approximately 5 h after drug administration. Sandwiches from a local deli were provided for lunch between scanning and the blood draw. Participants remained at the study site for approximately 6.5 h, either from 8:00 a.m.-2:30 p.m. or 10:00 a.m.-4:30 p.m., although all but two participants selected the late start time for all visits. A full timeline of events can be found in the supplement of Le et al.^46^.

### Analysis related to neuronally-enriched exosome and microRNA

#### Blood samples

Venous blood was collected in BD Vacutainer Serum Blood Collection tubes with spray-coated silica as a clot activator and then transported to the University of Oklahoma Integrative Immunology Center (IIC) within two hours of collection. Blood tubes were centrifuged at 1300×*g* for 10 min at room temperature, serum was removed, aliquoted, and then stored at − 80 °C until analysis.

#### Total exosomes (TEs) isolation

Total exosomes (TEs) were isolated from 250 µL (μL) serum samples using 63 μL of ExoQuick exosome precipitation solution (System Biosciences, CA, United States; Catalog #EXOQ5A-1). TE pellets were resuspended in 300 μL of 1X phosphate buffered saline (PBS) (Thermo Fisher Scientific, United States; Catalog #AM9625) with Halt protease and EDTA-free phosphatase inhibitor cocktail (Thermo Fisher Scientific, United States; Catalog #78425). TEs were used immediately or stored at − 80 °C until immunochemical enrichment of exosomes from neural sources could occur.

#### NEEs isolation

**Figure 7:**
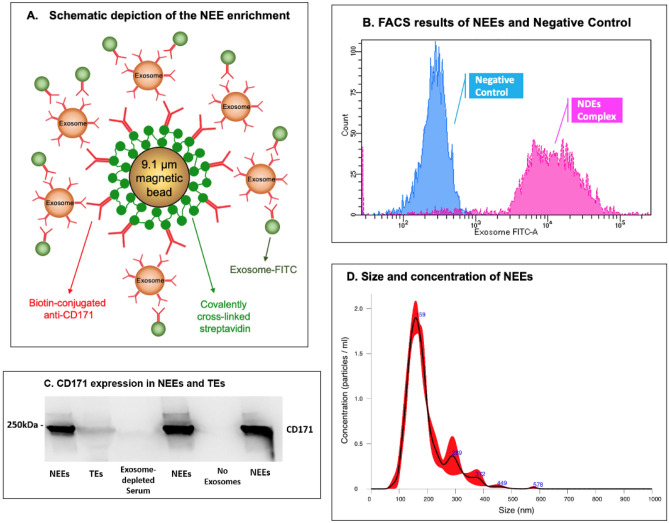
Neuronally-enriched exosomes (NEEs). (**A**) Schematic depiction of NEE enrichment. (**B**) Fluorescence activated cell sorting (FACS) results of NEEs and negative control (No exosomes). (**C**) Western blot analysis of NEEs, total exosomes (TEs), exosome-depleted serum, and negative controls (No exosomes) with anti-CD171 antibody marker, image cropped was from the same gel. (**D**) Size and concentration analysis of NEEs using Nanoparticle Tracking Analysis System.

TEs were enriched by a magnetic streptavidin bead immunocapture kit against the neural adhesion marker, L1CAM (CD171) biotinylated antibody (Fig. 7A). This technology to enrich NEEs in blood samples has been previously validated^47–49^. The CD171 (L1CAM, neural adhesion protein) marker was used for NEEs enrichment due to its high and relatively specific expression in neurons and low levels of expression in many other cell types (neuronal marker assessments show that majority of the exosomes in NEEs have a neuronal origin^47^, and the level of neuronal markers neurofilament-light (NF-L) and synaptophysin (SYP) are significantly enriched in NEEs by 86-fold and 951-fold compared to TEs^50^). Briefly, 80 µL of 9.1 µm diameter covalently cross-linked streptavidin magnetic beads (System Biosciences, CA, United States; Catalog #CSFLOWBASICA-1) and 80 µL of 100 nanograms/μL of mouse anti-human CD171 biotinylated antibody (clone 5G3, eBioscience, United States; Catalog #13-1719-82) were incubated on ice for 2 h with gentle flicking every 30 min. After washing three times in 1X Bead Wash Buffer (BWB) (Systems Biosciences, CA, United States; Catalog #CSFLOWBASICA-1) using a magnetic stand, the bead/antibody complex was suspended in 400 µL of BWB. 200 µL of TE suspensions were added to the bead/antibody complex and incubated overnight at 4 °C with rotation. After confirmation by flow cytometry, NEEs were eluted from the beads using 300 µL of Exosome Elution Buffer (System Biosciences, CA, United States; Catalog #CSFLOWBASICA-1), and NEEs were used immediately or stored at − 80 °C for miRNAs purification.

#### Flow cytometry

Once the NEEs were captured and stabilized, the bead/antibody/exosome complex was coupled to a fluorescein isothiocyanate (FITC) fluorescent tag (Exo-FITC, Systems Biosciences; Cat #CSFLOWBASICA-1) and subsequently analyzed by flow cytometry to confirm exosome capture as described by Winston et al.^51^ (Fig. 7A). The flow cytometric data were acquired using a BD LSR II Special Order Flow Cytometer, instrument performance was validated using BDTM Cytometer Setup and Tracking (CS&T) beads, and data were analyzed using FACS DIVA 8.0 software (BD Biosciences, San Jose, CA). Figure 7B shows an example of successful exosome capture using the beads coated with Exo-FITC antibodies specific for exosomes. Beads without exosomes were used as a negative control.

#### Western blot

Enrichment of NEEs from TEs was confirmed by western blot as described by Saeedi et al.^52^ (Fig. 7C). Briefly, electrophoresed gels were transferred unto polyvinylidene difluoride (PVDF) membranes using a Trans-Blot® Turbo transfer system. PVDF membranes were incubated with primary mouse antibody against CD171 (1:1000, CD171 Monoclonal Antibody (eBio5G3 (5G3), eBioscience™, Catalog # 13-1719-82) overnight at 4 °C. Following incubation with a horseradish peroxidase-conjugated anti-mouse antibody (1:2000, Cell Signaling, Catalog # 7076S) for 1 h, PVDF membranes were visualized by Clarity Max Western ECL Substrate (Bio-Rad, USA, Catalog # 1705062) and imaged using ImageQuant LAS 4000 (GE Healthcare Bio-Science, Sweden). The image displayed in Fig. 7C was from the same gel, the blot was not cut prior to hybridization with antibodies, full-length gel is included in Supplemental Fig. S1, the same full-length gel using different contrasts were in Supplemental Figs. S2 and S3.

#### Nanoparticle analysis

Size and concentration of NEEs were determined using Nanoparticle Tracking Analysis system (NanoSight NS300, Malvern Panalytical Inc., Malvern, United Kingdom). Figure 7D showed that the majority of captured NEEs were in the exosome size range, with mean size of 175 nm, and a standard deviation of 53 nm; the average concentration of NEEs was approximately 1.6 × 10^8^ particles per mL.

#### NEEs MiR purification

Purification was conducted using a Qiagen miRNeasy Micro Kit (QIAGEN, United States) according to the manufacturer’s protocol. Small RNA concentration was measured using an Agilent Small RNA kit (Agilent, United States) on a Bioanalyzer 2100 instrument (Agilent, United States). MiR samples were stored at − 80 °C until sequencing.

#### NEEs MiR sequencing and data processing

MiR samples were sent to the Oklahoma Medical Research Foundation (OMRF) Clinical Genomics Center for Next Generation Sequencing (NGS). MiR libraries were generated with a Qiagen QIAseq MiR library preparation kit and NGS was performed on an Illumina NextSeq HO SR75. Raw sequence FASTQ files received from OMRF were imported to Partek Flow software (https://www.partek.com/partek-flow/) for data analysis. Adapters from 3’ end were trimmed from the raw read after a quality check, and then aligned to the human genome hg38 using Bowtie alignment. Next, the aligned reads were quantified against the human miRbase mature microRNAs version 22 and reads from miR genes were normalized and scaled to reads per million for statistical data analysis.

### Neuroimaging

#### MID task

This task contained trials where participants saw a cue then a target, and the objective was to press a button as quickly as possible while the target was on the screen. Cues indicated the possible outcomes of a trial, with circle cues indicating a gain for hitting the target and square cues indicating a loss for missing the target. The magnitude of potential gain/loss was indicated by the position of a line on the cue and text showing the trial type (− 5/− 1/− 0/+ 0/+ 1/+ 5). There were 90 trials (15 of each condition) split across two 568 s runs. Target duration was calibrated based on a practice session completed before the scan and adjusted during scanning, so that on average participants hit on 60% of trials and earned $30 for the task.

fMRI data were acquired during the MID task using two identical GE MR750 3 T scanners using echo-planar imaging and the following parameters: 39 axial slices, TR/TE = 2000/27 ms, FOV/slice = 240/2.9 mm, 128 × 128 matrix. High-resolution structural images were obtained through a 3D axial T1-weighted magnetization-prepared rapid acquisition with gradient echo (MP-RAGE) sequence (TR/TE = 5/2.0 12 ms, FOV/slice = 240 × 192/0.9 mm, 186 axial slices). fMRI preprocessing was done using Analysis of Functional Neuro Imaging (AFNI)^53^ and consisted of: removal of the first three EPI volumes for signal stabilization, despiking, slice timing correction, co-registration to the anatomical image, motion correction via rigid-body alignment, and normalization to the Montreal Neurological Institute (MNI) standard space while resampling to 2 × 2 × 2-mm voxels, and smoothing with a 4-mm full-width at half-maximum filter. A general linear model was used to model the BOLD response during the anticipation phase of the MID with regressors for each of the 6 conditions (− 5/− 1/− 0/+ 0/+ 1/+ 5) as well as the six motion parameters and four polynomial terms. Voxelwise beta coefficients representing percent signal change were taken to the group level.

### Statistical analysis

#### Statistical analysis on NEEs MiR

Normalized MiR genes miR-27b-3p and miR-320b were log-transformed due to non-normality and used as the dependent variable in a repeated measure analysis of variance (ANOVA) with dose (placebo, ibuprofen 200 mg, ibuprofen 600 mg) as the within-subjects variable; paired t-tests were employed to test mean differences between doses. Similar repeated ANOVA tests and paired t-tests were also estimated for other MiRs with enough reads for statistical analysis.

#### MID analyses

3dLME^54^ was used to fit models with beta ~ miR*drug + visit (T1/T2/T3) + condition for gains and losses separately. Random effects of subject and visit nested within subject were included. Four models were run, for gains/losses and miR-27b-3p/miR-320b. After fitting each model, smoothness of the residuals was estimated using 3dFWHMx. Then 3dClustSim was used to estimate the family-wise error rate (FWER) given voxel-wise and cluster-size thresholds. Results are reported with a voxel-wise threshold of p < 0.001 and a FWER of α < 0.01. Effect sizes are reported based on the same LME models run post-hoc on average percent signal change in significant clusters.

## Supplementary Information

Supplementary Information.
