# Supplementary material for: Neuronally-enriched exosomal microRNA-27b mediates acute effects of ibuprofen on reward-related brain activity in healthy adults: a randomized, placebo-controlled, double-blind trial
Source: Sci Rep. 2022 Jan 17;12:861. doi: 10.1038/s41598-022-04875-y (PMC8764091; doi:10.1038/s41598-022-04875-y)
Supplement: Supplementary file 1 — Supplementary Information. [file 41598_2022_4875_MOESM1_ESM.docx]

***Supplemental Materials***

**Neuronally-enriched exosomal microRNA-27b mediates acute effects of ibuprofen on reward-related brain activity in healthy adults: a randomized, placebo-controlled, double-blind trial.**

Kaiping Burrows^1*^, Leandra K. Figueroa-Hall^1^, Rayus Kuplicki^1^, Jennifer L. Stewart^1,2^, Ahlam M. Alarbi^3^, Rajagopal Ramesh^4^, Jonathan B. Savitz^1,2^, T. Kent Teague^3,5,6^, Victoria B. Risbrough^7,8^, & Martin P. Paulus^1,2^

1. Laureate Institute for Brain Research, Tulsa, OK

2. Department of Community Medicine, University of Tulsa, Tulsa, OK

3. Departments of Surgery and Psychiatry, School of Community Medicine, The University of Oklahoma, Tulsa, OK

4.Department of Pathology, University of Oklahoma Health Sciences Center, Oklahoma City, OK

5. Department of Biochemistry and Microbiology, The Oklahoma State University Center for Health Sciences, Tulsa, OK

6. Department of Pharmaceutical Sciences, The University of Oklahoma College of Pharmacy, Oklahoma City, OK

7. Center of Excellence for Stress and Mental Health, La Jolla, CA

8. Department of Psychiatry, University of California, San Diego, La Jolla, CA

Corresponding author:

Kaiping Burrows, Ph.D.

Laureate Institute for Brain Research

6655 South Yale Ave, Tulsa, OK. 74136.

Email: [kburrows@laureateinstitute.org](mailto:kburrows@laureateinstitute.org)

**Tables S2** **through S5** list all clusters where either a significant main effect of miR or miR by condition interaction was detected with a voxelwise p < 0.001 and clusterwise α < 0.01. Bars in the Pattern column represent the slope relating miR to percent signal change at each dose of ibuprofen, computed by fitting a linear mixed effects model on the mean beta values extracted from each cluster. Bars are in the order Placebo, 200mg Ibuprofen, and 600mg ibuprofen, with beige indicating a positive slope (increased miR was related to increased activation) and blue indicating a negative slope (increased miR was related to decreased activation). For anticipation of gain trials, there was a significant interaction between condition and miR such that the relationship between miR and activation was attenuated by ibuprofen in 15 different clusters. In loss anticipation trials, there were several main effects of miR 27b-3p in four clusters such that higher level of MiR was associated with lower activation. Moreover, there were miR by condition interactions in 11 clusters, characterized by an enhanced negative association between miR and activation observed with the 600 mg dose of ibuprofen. Finally, miR-320b showed interactions in 8 mainly visual clusters during gain anticipation, and a main effect in one cluster during loss anticipation.

**Supplemental Table S1. NEE MiRs (n=20)**

|  | Placebo | Ibuprofen 200mg | Ibuprofen 600mg | Repeated ANOVA | 200mg vs. 0mg | 600mg vs. 0mg | 600mg vs. 200mg |
| --- | --- | --- | --- | --- | --- | --- | --- |
|  | *Mean (sd)* | *Mean (sd)* | *Mean (sd)* | *p-value*^b^ | *p-value*^c^ | *p-value*^c^ | *p-value*^c^ |
| ^a^let-7a-5p | 2.70 (0.90) | 2.73 (0.65) | 2.95 (0.55) | 0.155 | 0.854 | 0.152 | 0.076 |
| ^a^let-7b-5p | 3.45 (1.14) | 3.59 (0.92) | 3.73 (0.82) | 0.331 | 0.553 | 0.223 | 0.251 |
| ^a^let-7c-5p | 2.17 (0.73) | 2.43 (0.78) | 2.50 (0.63) | 0.153 | 0.113 | 0.060 | 0.664 |
| ^a^let-7f-5p | 2.94 (1.22) | 3.18 (0.80) | 3.25 (0.56) | 0.529 | 0.319 | 0.254 | 0.633 |
| ^a^let-7i-5p | 2.51 (1.28) | 2.52 (0.97) | 2.48 (0.85) | 0.983 | 0.970 | 0.928 | 0.854 |
| ^a^miR-1-3p | 2.88 (0.96) | 3.18 (0.70) | 3.18 (0.73) | 0.537 | 0.321 | 0.308 | 0.986 |
| ^a^miR-122-5p | 1.38 (1.06) | 1.74 (1.22) | 1.47 (0.95) | 0.394 | 0.197 | 0.728 | 0.290 |
| ^a^miR-126-3p | 2.67 (1.46) | 2.68 (1.27) | 2.83 (0.87) | 0.735 | 0.979 | 0.629 | 0.552 |
| ^a^miR-142-3p | 2.52 (1.25) | 2.34 (1.09) | 2.53 (0.95) | 0.676 | 0.637 | 0.982 | 0.379 |
| ^a^miR-146a-5p | 1.45 (0.92) | 1.36 (1.01) | 1.39 (0.90) | 0.962 | 0.778 | 0.844 | 0.889 |
| ^a^miR-16-5p | 4.45 (1.61) | 4.42 (1.35) | 4.61 (0.87) | 0.605 | 0.958 | 0.530 | 0.482 |
| ^a^miR-184 | 2.67 (0.88) | 3.09 (0.92) | 3.36 (1.12) | 0.059 | 0.097 | 0.037 | 0.468 |
| ^a^miR-191-5p | 1.77 (0.91) | 1.71 (0.93) | 1.66 (0.83) | 0.924 | 0.816 | 0.688 | 0.824 |
| ^a^miR-203a-3p | 4.60 (1.28) | 4.99 (0.61) | 5.25 (0.55) | **0.040** | 0.224 | **0.037** | 0.069 |
| ^a^miR-203b-5p | 4.02 (1.31) | 4.16 (1.30) | 4.55 (0.98) | 0.134 | 0.745 | 0.129 | 0.160 |
| ^a^miR-205-5p | 2.74 (1.16) | 3.15 (0.70) | 3.29 (0.76) | 0.132 | 0.202 | 0.054 | 0.441 |
| ^a^miR-21-5p | 2.00 (0.93) | 2.46 (0.97) | 2.28 (0.75) | 0.232 | 0.086 | 0.236 | 0.449 |
| ^a^miR-221-3p | 1.06 (0.97) | 1.34 (0.87) | 1.44 (0.65) | 0.289 | 0.306 | 0.113 | 0.714 |
| ^a^miR-223-3p | 2.00 (1.14) | 1.66 (1.08) | 2.05 (0.87) | 0.265 | 0.287 | 0.851 | 0.099 |
| ^a^miR-23b-3p | 3.54 (1.14) | 3.63 (0.82) | 4.09 (0.70) | **0.010** | 0.791 | **0.041** | **0.017** |
| ^a^miR-26a-5p | 3.20 (1.19) | 3.38 (0.74) | 3.64 (0.67) | 0.130 | 0.514 | 0.116 | 0.079 |
| ^a^miR-26b-5p | 1.30 (0.92) | 1.31 (0.86) | 1.38 (0.83) | 0.929 | 0.986 | 0.734 | 0.719 |
| ^a^miR-27b-3p | 2.41 (0.94) | 2.68 (0.75) | 2.99 (0.59) | **0.015** | 0.306 | **0.014** | **0.046** |
| ^a^miR-320b | 1.27 (0.80) | 1.53 (0.87) | 1.91 (0.82) | **0.007** | 0.219 | **0.002** | **0.048** |
| ^a^miR-451a | 2.43 (1.32) | 2.13 (1.35) | 2.44 (1.01) | 0.515 | 0.429 | 0.955 | 0.259 |
| ^a^miR-92a-3p | 1.67 (0.96) | 1.66 (1.18) | 1.49 (0.88) | 0.708 | 0.986 | 0.464 | 0.545 |
| ^a^miR-9985 | 1.26 (0.73) | 1.27 (0.76) | 1.66 (0.71) | 0.063 | 0.972 | 0.055 | 0.052 |

Note. ^a^ Log-transformed; ^b^p-values are from repeated ANOVA; ^c^*p*-values are from paired t-test.

**Supplemental Table S2. miR-27b-3p Effects During MID Gain Anticipation. The graphical patterns represent the marginal effects of each dose (placebo, ibuprofen 200mg, and ibuprofen 600mg)**

| Effect | #Voxels | CM x | CM y | CM z | Alpha | Region | Pattern |
| --- | --- | --- | --- | --- | --- | --- | --- |
| Interaction | 210 | 36.7 | -48.3 | 16.4 | <<0.01 | Left Middle Frontal Gyrus | 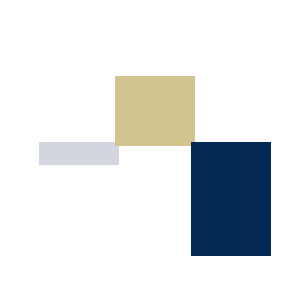 |
| Interaction | 188 | 2 | 78.1 | -1.5 | <<0.01 | Left Lingual Gyrus | 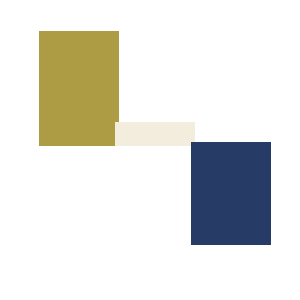 |
| Interaction | 146 | 11 | 70.3 | 22 | <<0.01 | Left Cuneus | 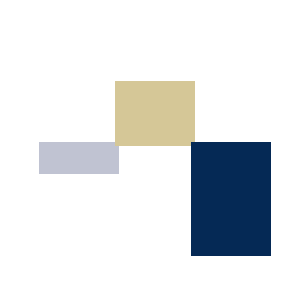 |
| Interaction | 132 | -33.5 | 52.5 | 57 | <<0.01 | Right Superior Parietal Lobule | 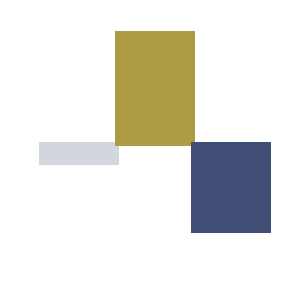 |
| Interaction | 125 | -42.7 | -48.7 | 8.5 | <<0.01 | Right Middle Frontal Gyrus | 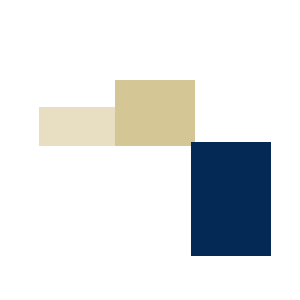 |
| Interaction | 116 | 5.5 | -37.3 | 56.5 | <0.01 | Left Superior Medial Gyrus | 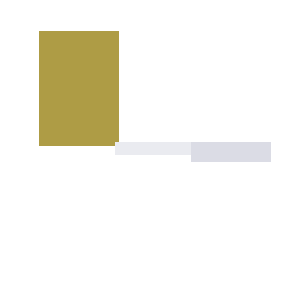 |
| Interaction | 97 | 38.2 | 74 | 27.8 | <0.01 | Left Middle Occipital Gyrus | 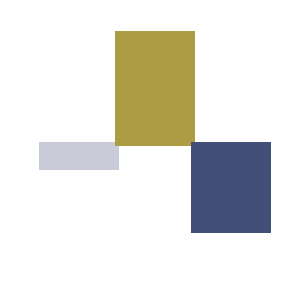 |
| Interaction | 89 | 31.5 | 6.1 | -2.9 | <0.01 | Left Putamen | 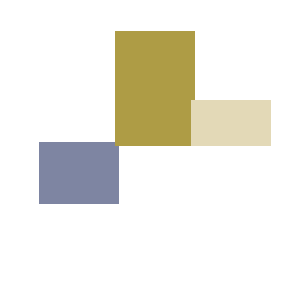 |
| Interaction | 83 | -19.3 | 71.8 | -1.4 | <0.01 | Right Lingual Gyrus | 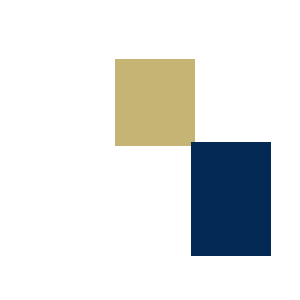 |
| Interaction | 82 | -48.2 | 66 | 18.9 | <0.01 | Right Middle Temporal Gyrus | 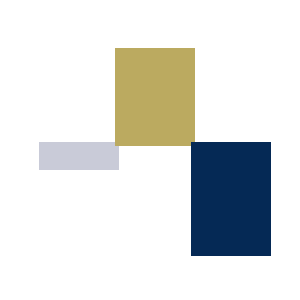 |
| Interaction | 81 | -5.4 | 35.5 | -26.1 | <0.01 | Brain-Stem | 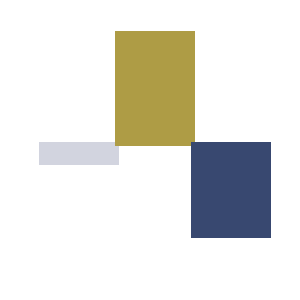 |
| Interaction | 80 | -29.1 | -2.7 | 1.2 | <0.01 | Right Putamen | 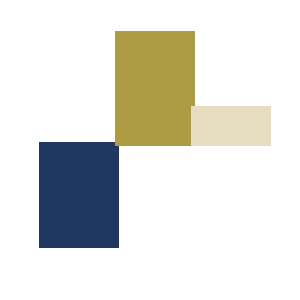 |
| Interaction | 67 | 13.4 | 81.7 | 27.4 | <0.01 | Left Superior Occipital Gyrus | 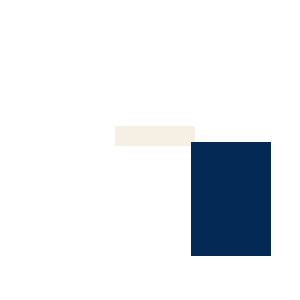 |
| Interaction | 62 | -13.4 | 80.8 | 33.6 | <0.01 | Right Cuneus | 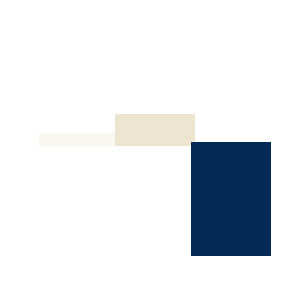 |
| Interaction | 61 | -25 | 68.9 | -27 | <0.01 | Right Cerebellum | 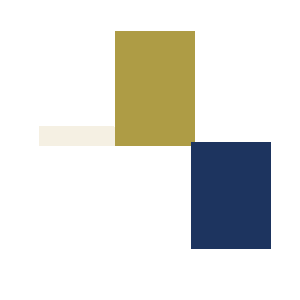 |

**Supplemental Table S3. miR-27b-3p Effects During MID Loss Anticipation. The graphical patterns represent the marginal effects of each dose (placebo, ibuprofen 200mg, and ibuprofen 600mg)**

| Effect | #Voxels | CM x | CM y | CM z | Alpha | Region | Pattern |
| --- | --- | --- | --- | --- | --- | --- | --- |
| Main | 181 | 11 | 43 | -3 | <<0.01 | Left Lingual Gyrus | 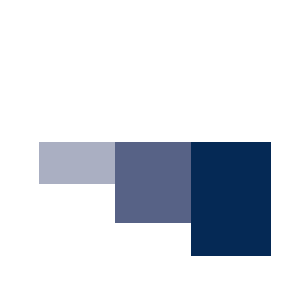 |
| Main | 176 | -27 | 53 | 9 | <<0.01 | Right Calcarine Gyrus | 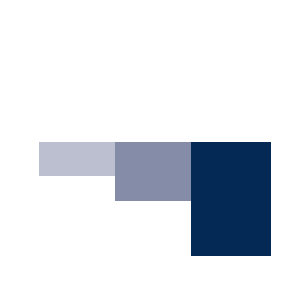 |
| Main | 156 | 41 | -9 | -15 | <<0.01 | Left Insula | 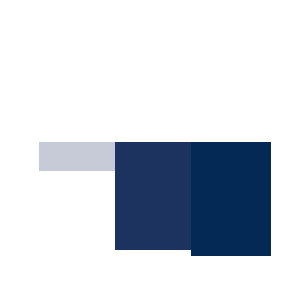 |
| Main | 62 | -41 | -5 | -11 | <0.01 | Right Insula | 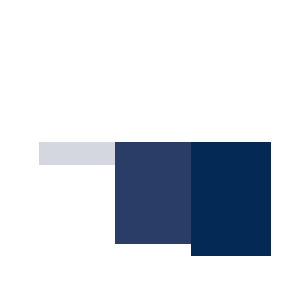 |
| Interaction | 243 | 60.8 | 11.5 | 9.3 | <<0.01 | Left Superior Temporal Gyrus | 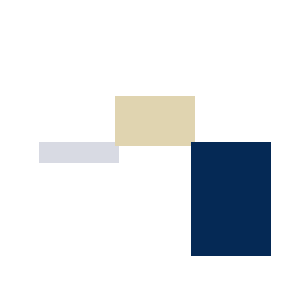 |
| Interaction | 222 | -6.9 | 72.9 | -3.1 | <<0.01 | Right Lingual Gyrus | 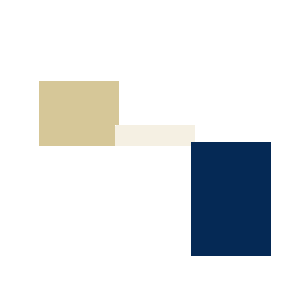 |
| Interaction | 214 | -57.1 | 6.3 | 11.1 | <<0.01 | Right Rolandic Operculum | 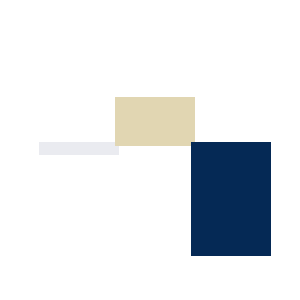 |
| Interaction | 105 | -48.7 | 31.5 | 17.4 | <0.01 | Right Superior Temporal Gyrus | 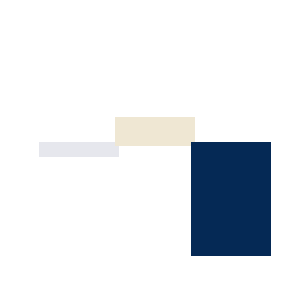 |
| Interaction | 99 | -9.3 | 77.2 | 32.3 | <0.01 | Right Cuneus | 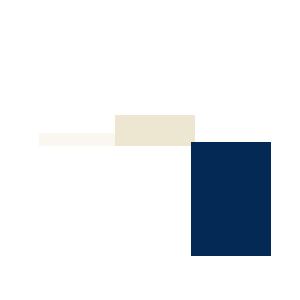 |
| Interaction | 76 | 26.3 | 68.2 | -9 | <0.01 | Left Fusiform Gyrus | 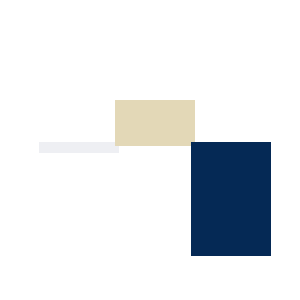 |
| Interaction | 73 | -9.1 | -24.4 | 8.3 | <0.01 | Right Cerebral White Matter | 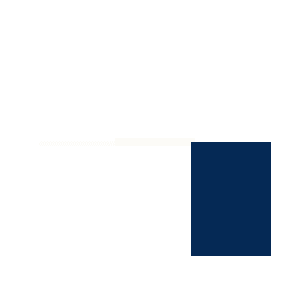 |
| Interaction | 73 | 14.1 | 68.2 | 20.9 | <0.01 | Left Cuneus | 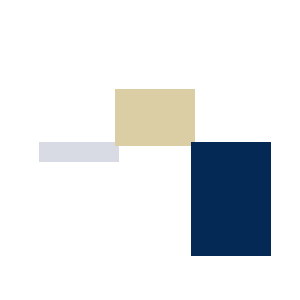 |
| Interaction | 69 | 11.1 | 63.3 | -1.1 | <0.01 | Left Lingual Gyrus | 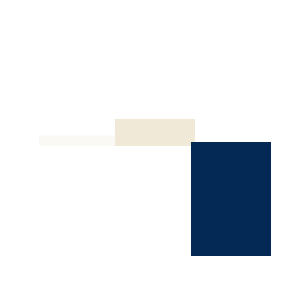 |
| Interaction | 68 | 12.8 | 84.3 | 29 | <0.01 | Left Cuneus | 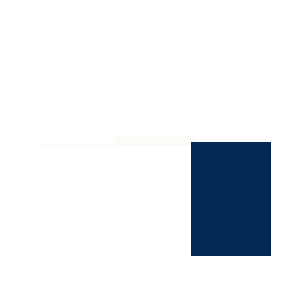 |
| Interaction | 63 | -61.7 | 29.6 | -2.3 | <0.01 | Right Middle Temporal Gyrus | 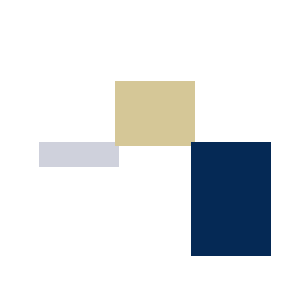 |

**Supplemental Table S4. miR-320b Effects During MID Gain Anticipation. The graphical patterns represent the marginal effects of each dose (placebo, ibuprofen 200mg, and ibuprofen 600mg)**

| Effect | #Voxels | CM x | CM y | CM z | Alpha | Region | Pattern |
| --- | --- | --- | --- | --- | --- | --- | --- |
| Interaction | 270 | -10.4 | 81 | -6.9 | <<0.01 | R Fusiform Gyrus | 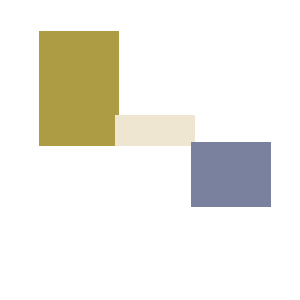 |
| Interaction | 224 | -48.7 | 64.3 | 17 | <<0.01 | R Middle Temporal Gyrus | 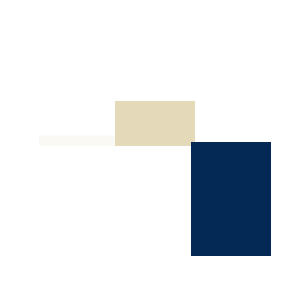 |
| Interaction | 115 | 14.5 | 66.9 | 51.1 | <0.01 | L Superior Parietal Lobule | 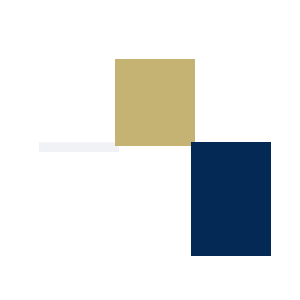 |
| Interaction | 111 | -12.3 | 74 | 47 | <0.01 | R Precuneus | 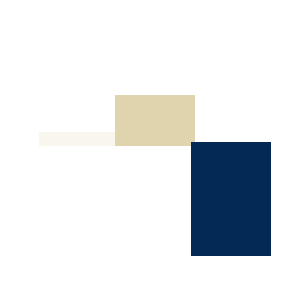 |
| Interaction | 83 | 11.3 | 86.2 | 30.2 | <0.01 | L Cuneus | 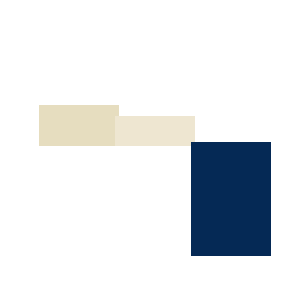 |
| Interaction | 77 | 39.2 | 78.2 | 29.5 | <0.01 | L Middle Occipital Gyrus | 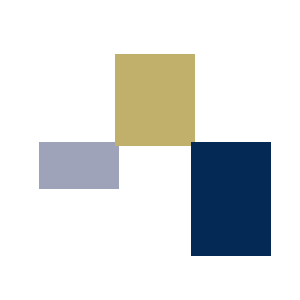 |
| Interaction | 75 | -35.2 | 45.3 | 49.7 | <0.01 | R Inferior Parietal Lobule | 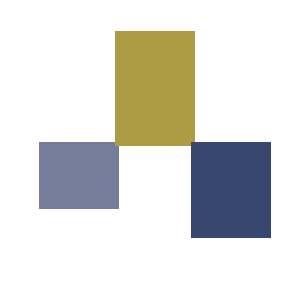 |
| Interaction | 70 | 29.2 | 70.7 | -28.9 | <0.01 | L Cerebellum | 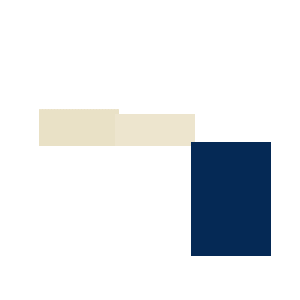 |

**Supplemental Table S5. miR-320b Effects During MID Loss Anticipation. The graphical patterns represent the marginal effects of each dose (placebo, ibuprofen 200mg, and ibuprofen 600mg)**

| Effect | #Voxels | CM x | CM y | CM z | Alpha | Region | Pattern |
| --- | --- | --- | --- | --- | --- | --- | --- |
| Main | 88 | 17.7 | 53.8 | 26.7 | <0.01 | Left Superior Frontal Gyrus | 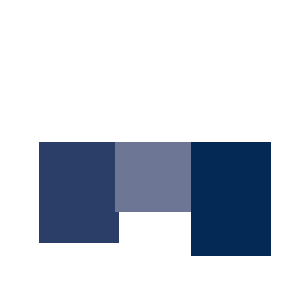 |

**Supplemental Figure S1 through S3** list original uncropped blot with multiple exposure images for illustrative Western Blot used in Figure 4C.

**Supplemental Figure S1. Original uncropped blot for illustrative Western Blot used in Figure 4C. The edges of the blot were outlined with black lines.**

**
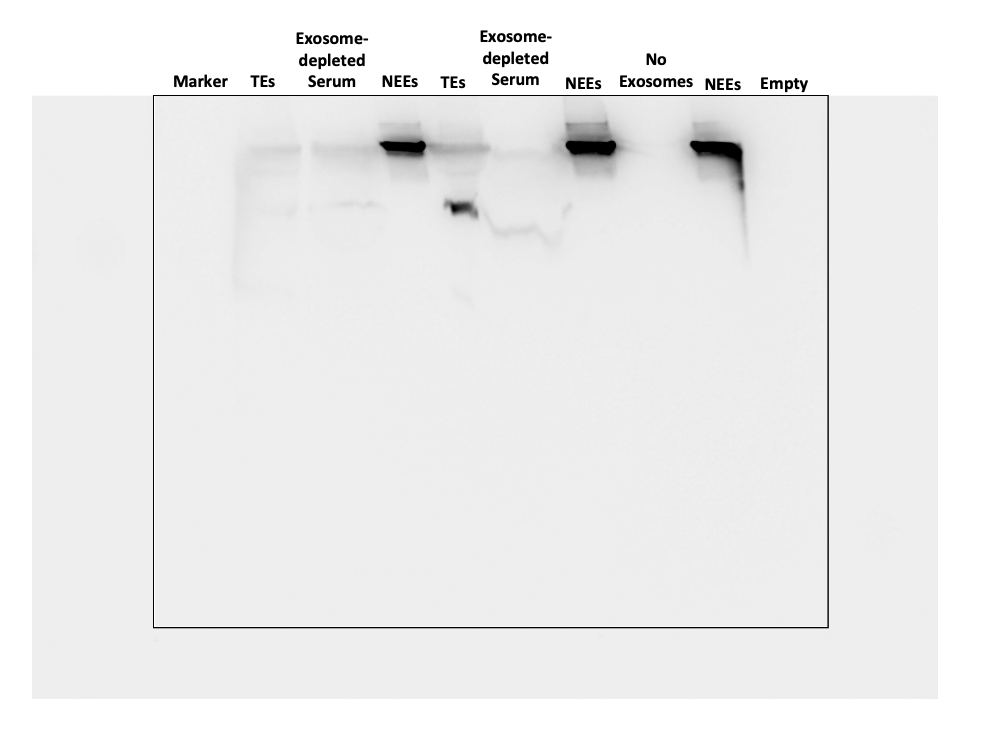
**

**Supplemental Figure S2. Original uncropped blot using different contrast for illustrative Western Blot used in Figure 4C. The edges of the blot were outlined with black lines.**

**
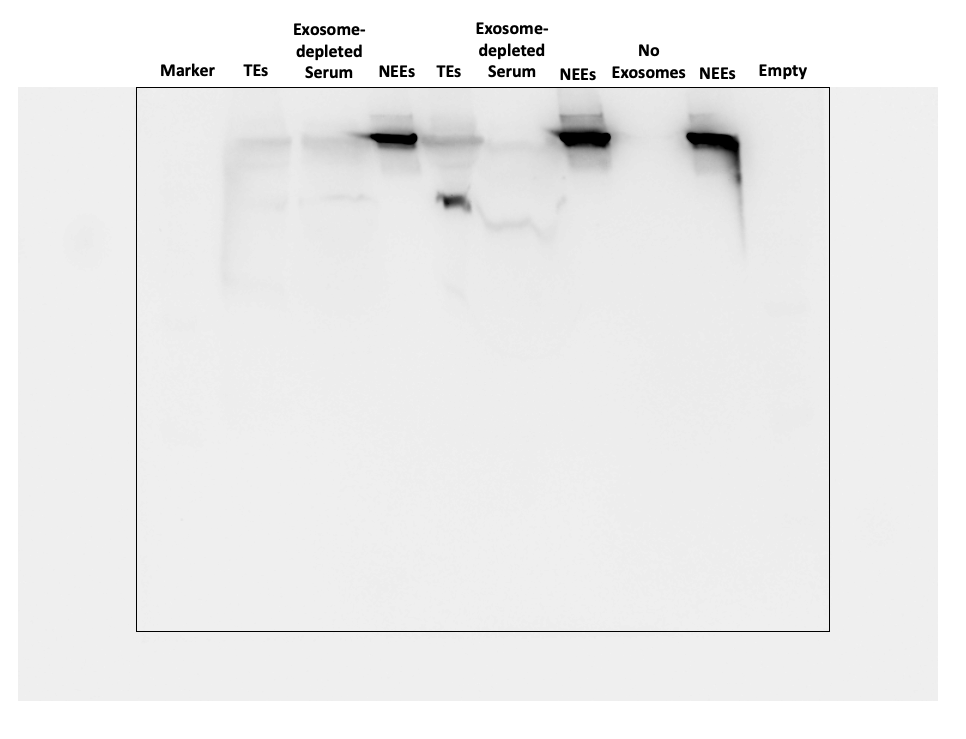
**

**Supplemental Figure S3. Original uncropped blot using different contrast for illustrative Western Blot used in Figure 4C. The edges of the blot were outlined with white lines.**

**
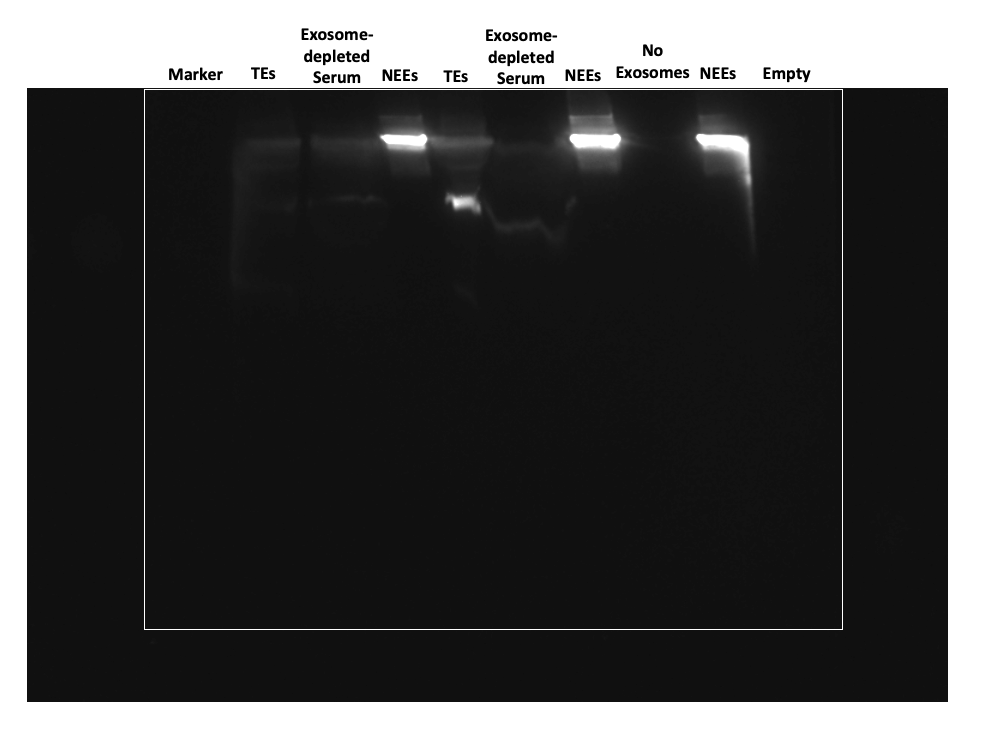
**
